# Tuning the dimensionality of ZnO nanowires through thermal treatment: An investigation of growth mechanism

**DOI:** 10.1186/1556-276X-7-354

**Published:** 2012-06-28

**Authors:** Po-Hsun Shih, Hsuan-Jung Hung, Yuan-Ron Ma, Sheng-Yun Wu

**Affiliations:** 1 Department of Physics, National Dong Hwa University, Hualien, 97401, Taiwan

**Keywords:** nanocrystalline materials, short-circuit diffusion, lattice diffusion, nanowires, ZnO, 61.46.Hk, 61.82.Fk, 62.23.Hj, 66.30.Pa

## Abstract

In this study, we synthesized various dimensionalities of ZnO nanowires using the Ti grid-assisted chemical vapor deposition process. Energy dispersive X-ray spectroscopic mapping technique accompanied with a lattice diffusion model was used to characterize the growth mechanism. A diffusion ratio γ, defined by short-circuit and lattice diffusion activation energies, was obtained to describe the growth mechanism of ZnO nanowires. The tunable dimensionalities of ZnO nanowires allow us to modify the morphology of ZnO nanocrystals by developing well-controlled potential applications.

## Background

Dense arrays of oriented, crystalline ZnO nanowires have attracted much attention for applications in nanoscale lasers [1], light-emitting diodes [2,3], sensors [4], and solar cells [5]. Dimensionality and size are the two key factors that govern the properties of nanostructures affected by their high surface-to-volume ratio [6,7]. The requirements for dimensional control, especially of size and morphology effects on nanoparticles, nanowires, and three-dimensional (3-D) nanowires, seem to still be a challenge. Some 3-D nanostructural materials have been synthesized. These works have primarily focused on the synthesis of inorganic [8], polymeric nanomaterials [9,10], and dendrite nanowires [11,12]. To date, ZnO has displayed a series of nanostructures with different morphologies or dimensionalities [13,14]. It is believed that the discrepancy of the formation under different growing methods is vitally responsible for shape modifications of ZnO. Understanding the relation between growth mechanism and dimensionality is attracting more attention than ever before and is becoming urgently important for obtaining nanowires with a desired size, shape, and dimensionality. However, exact control for growing multidimensional-oriented arrays of ZnO still remains out of reach. The development of a simple, easily controllable method for growing one-dimensional (1-D) to 3-D ZnO nanowire arrays is of great significance [15,16]. In this study, we have successfully synthesized well-separated one- to three-dimensional ZnO nanowire networks using a Ti grid-assisted thermal evaporation approach. Energy dispersive X-ray spectroscopic (EDS) mapping was used to investigate the scale structure of ZnO nanowire. The formation of various dimensional ZnO nanowires is attributed to short-circuit and lattice diffusion mechanisms.

## Methods

**Figure 1:**
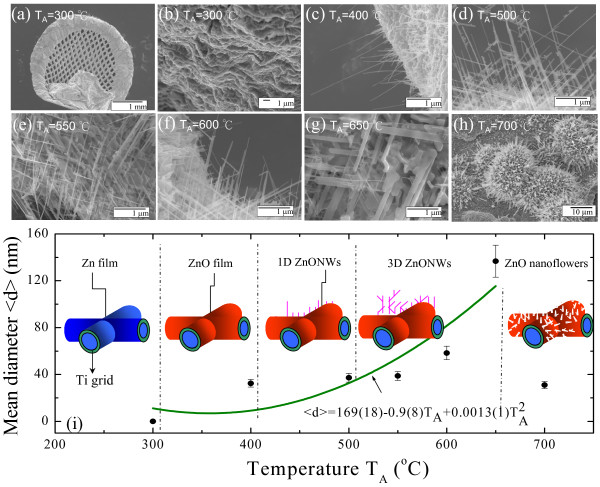
**Annealing temperature dependence of SEM images and <** ***d*** **>.** (**a**) Low- and (**b**) high-magnification SEM images of the ZnO film on Ti grid at *T*_A_ = 300°C. SEM images of ZnO nanocrystals after various annealing temperatures with (**c**) 1-D ZnO nanowires at *T*_A_ = 400°C; (**d**) to (**g**) 3-D ZnO nanowires at *T*_A_ = 500°C, 550°C, 600°C, and 650°C, respectively; (**h**) ZnO nanoflowers at *T*_A_ = 700°C; (**i**) the growth temperature *T*_A_ dependence of the mean diameter <*d*> obtained from a portion of an SEM image of ZnO nanowires, where the solid curve is a guide for the eye.

A series of three-dimensional ZnO nanowire network was fabricated [17]. The samples were prepared by a process where a pure zinc ingot was mounted in a Ti hollow grid which was then placed in a ceramic boat inside a quartz tube. The tube was evacuated to about 10^−3^ Torr using a mechanical pump and heated in a tube furnace at about 200°C for 2 h to form a Zn film. The Ti grid samples with the resultant Zn film were then heated to various annealing temperatures *T*_A_ ranging from 300°C to 700°C for 2 h in a mixed argon (100 sccm) and oxygen (10 sccm) atmosphere. The dimensionality and morphology of various ZnO nanocrystals are shown in Figure 1a,b,c,d,e,f,g,h. Details of the shape and morphology of the prepared nanocrystals were characterized using field-emission scanning electron microscopy (FESEM; JEOL JSM-6500 F, JEOL Ltd., Akishima, Tokyo, Japan). Figure 1a,b,c,d,e,f,g,h shows typical images of the SEM morphology on the as-synthesized products at various *T*_A_. It can be seen that the ZnO grew homogeneously in a large area of the Ti grid substrate to form a ZnO film at *T*_A_ = 300°C, well-separated straight nanowires at *T*_A_ = 400°C, three-dimensional ZnO nanowires between *T*_A_ = 500°C to 650°C, and ZnO nanoflowers at *T*_A_ = 700°C.

As shown in Figure 1c, the as-grown 1-D ZnO nanowires grew homogeneously on the Ti grid substrate to form straight nanowires. Observation of the uniform nanowires (with lateral dimensions, on the order of nanometers, i.e., in the hundred to ten nanoscale) shows that they grew up to a few microns in length. By controlling the growth temperature *T*_A_ = 500°C to 650°C, the 3-D hybrid ZnO nanostructure is seen in Figure 1d,e,f,g. Also, the average diameter estimated from the main arms increases with *T*_A_ increase. As a result, shown in Figure 1h, the separated 3-D ZnO nanowire network finally grow into 2-D nanosheets through the high-temperature process, aggregating and leading to form nanosheet-assembled ZnO flowers. The mean ZnO nanowire diameters <*d*>, as determined from the SEM images (Figure 1c,d,e,f,g,h) and described by the fit of the log-normal functions,^a^ were approximately 32(3), 37(4), 38(4), 58(6), 131(13) and 31(3) nm, respectively. The diameter of a ZnONW ranges from 32 to 131 nm. The length of the ZnONWs was found to be dependent on the deposition time and annealing temperature. It is worth noting that the present size of ZnO nanoflowers is defined from the distribution of the mean width of each nanosheet.

The *T*_A_ dependence of the mean diameters obtained from a portion of the SEM image is shown in Figure 1i, where the solid curve shows the fit to the growth law. This can be well described as <*d*> = 169(19) − 0.9(8)*T*_A_ + 0.0013(1)*T*_A_^2^. In this present study, the growth temperature of ZnO nanowires was confined to between 400°C to 650°C, which is 0.21 and 0.33 times the melting point of ZnO (1,975°C), following the Wagner's scaling theory [18]. This result is different with the previous report by Jeong group that claimed higher annealing temperature results in lower dimensionality in ZnO nanowire using MOCVD method [19]. In case of the MOCVD, the growth of ZnO nanowires could be explained by the island formation on compressively strained sites and surface diffusion of source materials at supersaturation level. In the present case of the CVD, the diffusion of Zn on the surface, it is necessary to consider the thermal energy and initial growth into wire-like structure that are kinetically favored. Thereby, detailed studies of size and morphology of ZnO nanowires may greatly contribute to the understanding of growth mechanism.

## Results and discussion

### Analysis of crystal structure by TEM

**Figure 2:**
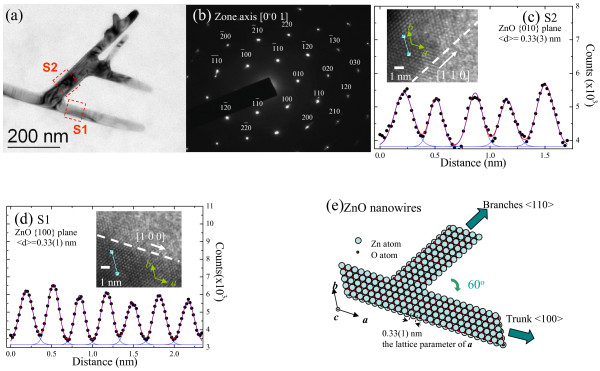
**TEM image, electron diffraction pattern, height-position information, and schematic plot.** (**a**) High-magnification bright-field TEM image revealing the dendritic ZnO structure. Many short branches extending from the side of the main arm can be observed. (**b**) Electron diffraction pattern of the selected area of the sample, revealing the [0 0 1] zone axis of the ZnO nanowires. (**c**) to (**d**) The resulting height-position information for two selected perpendicular section lines taken from HRTEM images of a high-magnification enlargement of the selected region marked in Figure 2a. (**e**) Schematic plot of a single dendritic ZnO nanowire structure.

Transmission electron microscopic (TEM), selected area electron diffraction (SAED) pattern, and high-resolution transmission electron microscopic (HRTEM) images from a JEM-3010 transmission electron microscope (JEOL Ltd., Japan) were obtained to study the crystalline structure. As an example, we show the case of 3-D ZnO nanowires with crystalline structures. Figure 2a shows the TEM morphology of a typical 3-D ZnO nanowire. This TEM image reveals that most of the nanowires are straight, and the diameter along the growth direction is uniform, with a mean diameter of 43(1) nm for the main arms (marked as S1) and 55(2) nm for the branches (marked as S2). The single crystalline nature of the sample studied is clearly revealed. The Bragg spots correspond to the zone axis [0 0 1] reflection of the wurtzite structure of the ZnO. Shown in Figure 2b is the SAED result; the pattern of the main spots can easily be seen appearing as hexagonal cells with lattice parameters of *a* = 3.250(5) Å and *c* = 5.205(3) Å, which indicate a predominantly crystalline hexagonal wurtzite ZnO. The detailed structure of the controlled-growth 3-D ZnO nanowires was further investigated using HRTEM. Two high-magnification enlargements of a selected region of the HRTEM images in Figure 2a at branched regions are shown. Figure 2c,d shows an enlargement along two selected perpendicular section lines with the corresponding HRTEM images shown in the insets. Gray-level analysis of the image is employed to extract height information. The resulting height-position information is fitted using Gaussian functions to obtain the average atomic spacing. The fitted inter-planar distances of the two fingers are 0.33(1) (marked S1) and 0.33(3) nm (marked S2) corresponding to the [1 0 0] and [0 1 0] planes of the ZnO nanowire, respectively. The results are plotted schematically in Figure 2e. It can be seen that for the growth direction of these two branches is indicated by [1 0 0] and [1 1 0], and they are inclined towards each other by 60° at the boundary regions, resembling in form the dendritic 3-D ZnO nanowires. The growth mechanism of ZnO nanocrystals was reported in the previous work by Fan group [12]; they explained and proposed that the growth of the main arms and branches is through a self-catalytic liquid–solid and vapor-solid processes, respectively. However, in the multidimensional ZnO nanowire system, the growth process is dependent on the surface diffusion of Zn, with respect to the annealing temperature and time. To verify the formation of ZnO nanowires, further SEM investigation was carried out.

### EDS mapping

**Figure 3:**
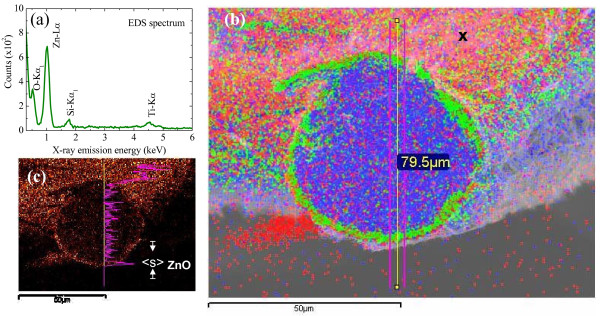
**Typical EDS spectrum, two-dimensional EDS mapping images, and line profile EDS analysis.** (**a**) Typical EDS spectrum revealing the elemental composition of the selected sample at *T*_A_ = 400°C. (**b**) Two-dimensional EDS mapping images of the distribution of elements presented using the lock-in energy of Ti-Kα (4.3 to 4.7 keV), O-Kα_1_ (0.4 to 0.6 keV), and Zn-Lα (0.8 to 1.2 eV). (**c**) Line profile EDS analysis clearly showing the presence of O in the sample. The line width of the distribution can be used to define the mean diffusion length <*s*>.

An EDS (Oxford Instruments Inca x-sight model 7557, Abingdon, Oxfordshire, UK) mapping technique was used to measure the surface thickness of the ZnO film on the Ti grid. EDS mapping generates a two-dimensional image indicating the abundance of an element. The intensity of the image allows direct visualization of the spatial distribution of any element, such as zinc, titanium, or oxygen, on the surface of the Ti grid. The typical EDS elemental spectrum taken at the surface of the Ti grid is shown in Figure 3a, where the peaks are associated with a series of elemental O, Zn, Si, and Ti, which can be assigned to O-Kα_1_, Zn-Lα, Si-Kα_1_ (Si substrate from mounting the sample) and Ti-Kα. Moreover, the Zn/O ratio is estimated to be 1.05(5), which is close to the stoichiometric composition of ZnO, indicating the high purity of the nanowires. Figure 3b depicts an EDS mapping of a cross section of the Ti grid of the selected sample (at *T*_A_ = 400°C), where the distribution of elements is presented using the lock-in energy of Ti-Kα (4.3 to 4.7 keV), O-Kα_1_ (0.4 to 0.6 keV), and Zn-Lα (0.8 to 1.2 eV), respectively. The formation of ZnO nanowires can be mapped by EDS observations through cross section of the Ti grid. It can be seen that the core is dominated by Zn that diffuses into the hollow Ti grid to form a Zn core (blue color), Ti shell (green color), and surface ZnO film (orange color), respectively. The line profile EDS analysis extracted from EDS mapping clearly shows the distribution of oxygen in Figure 3c. As shown in Figure 3c, the width of the ZnO thickness <*s*> can be used to estimate the length of diffusion of the zinc at various *T*_A_ and time *τ*. Details related to the estimating length are listed in Table 1. Thermal treatment of the Zn on the Ti grid is known to influence the rates of oxide growth during nucleation and nanowire formation. The diffusion length is also sensitive to the thermal treatment time. A diffusion model is employed to interpret the oxidation kinetics wherein zinc transport proceeds in ZnO both by short-circuit and lattice diffusions.

**Table 1:** **ZnO thickness <*s*> along with simulated results**

***T***_**A**_**(K)**	**<** ***s *> (μm)**	***γ***	***Q***_**S**_**= *γQ***_**D**_**(kJ/mol)**
673	6.78(1)	0.26(6)	80
773	4.21 (5)	0.31(2)	99
873	3.23(3)	0.36(3)	114
973	2.72(5)	0.41(3)	130

Results for the lattice and short-circuit diffusion theories over the complete range of annealing temperatures, where *Q*_S_ is the short-circuit activation energy. Lattice activation energy *Q*_D_ = 318 kJ/mol [20].

### Short-circuit diffusion in ZnO nanowires

**Figure 4:**
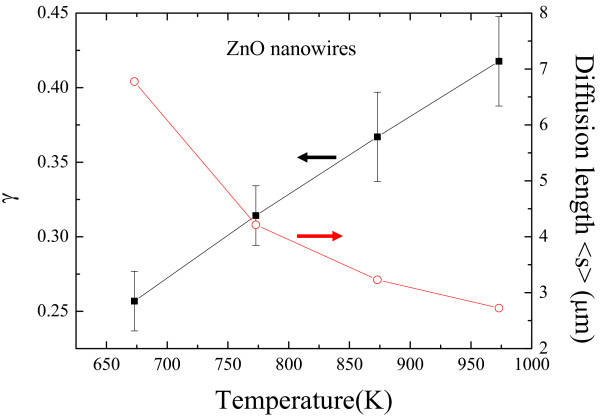
**Plots of diffusion ratio *γ *dependence on the annealing temperature for ZnO nanowire together with <*s*>.**

In case of ZnO, where the diffusion coefficient of zinc is higher than that of oxygen, lattice diffusion takes place, resulting in an increase in the forming of ZnO with annealing temperature. It is well known that annealing of the Zn at a temperature above 300°C results in parabolic growth of ZnO nanocrystals (seen in Figure 1)i. The contribution from boundary diffusion at the grain boundaries decreases with increasing nanocrystal size [21,22]. It is therefore important to consider the influence of the scale on zinc transport when metal facets are oxidized at temperatures less than one-half the melting point of ZnO. At these temperatures, recrystallization and grain growth proceed slowly, with polycrystalline oxide boundaries serving as effective short-circuit diffusion paths [23]. A simple diffusion model is employed to interpret the oxidation kinetics. In this model, Zn transport proceeds in ZnO both by short-circuit diffusion at the grain boundaries and by lattice diffusion at higher annealing temperatures. The theory of lattice diffusion fully explains the diffusion mechanism [20,24-26]. In order to clarify the contribution from short-circuit or lattice diffusion, we have defined a diffusion ratio *γ* taken as the percentage of lattice activation energy of Zn ions (where *γ* = 1/3 and 1 indicates short-circuit or lattice diffusion, respectively). The estimated thickness <*s*> is related to the diffusion length $\sqrt{D_{\text{Zn}}^{*}\cdot \tau }$ that can be obtained from the lattice diffusivity $D_{\text{Zn}}^{*}\left({\text{cm}}^{2}{\text{s}}^{-1}\right)$ utilizing the following formula:

(1) $$D_{\text{Zn}}^{*}=\frac{\beta }{2}\alpha^{2}v_{\text{D}}\exp\left\{-\frac{\gamma Q_{\text{D}}}{RT_{\text{A}}}\right\}\text{,}$$

where *β* = 2 is the number of positions a Zn atom can jump along the [1 0 0] plane; *α* = 0.285 nm, the *d*-spacing of the [1 0 0] plane; *v*_D_ 1.73 × 10^11^ s^−1^, the vibration frequency;^b^*τ*, the growth time (approximately 7,200 s); *Q*_D_ = 318 kJ/mol, the activation energy of Zn [27]; *R* (1.987 cal mol K^−1^), the gas constant; and *T*_A_ (K), the annealing temperature. The resultant diffusion ratio *γ* dependence on the annealing temperature for ZnO nanowire, together with < *s* >, was shown in Figure 4. Details related to the fitting parameters are listed in Table 1. The *γ* value of approximately 0.26(6) is slightly lower than the short-circuit diffusion predicted value of approximately 1/3 at lower *T*_A_ = 400°C, revealing that the growth of nanowires is influenced by grain boundaries, whether oxygen or zinc is the diffusion species along short-circuit path dislocations, resulting in the growth of 1-D ZnO nanowalls at the lower temperature regime. These characteristics agree with previously reported for a single CuO nanowire that occurs through the short-circuit diffusion mechanism obtained by Cheng et al. [25]. At higher *T*_A_, corresponding to our experimental data, the value of *γ* is close to 0.41(3), indicating that lattice diffusion is the dominant transport mechanism in the oxidation of Zn that forms a 3-D ZnO nanowire network.

In general, at high temperatures where the difference between lattice diffusivity and short-circuit diffusivity is relatively small, but at low temperatures, such at 400°C, the short-circuit diffusivity is many orders of magnitude greater than lattice diffusivity. Short-circuit diffusion makes the greatest contribution to the net flux (mass transport through a unit area) for the growth of nanowires at low temperatures. In our previous study [17], at lower *T*_A_ (approximately 400°C), it reveals that it is likely that the nanowire formation proceeds through the nucleation of ZnO_*x*_ and ZnO and that a boundary transition occurred during the growth process. The formation of a single ZnO nanowire is attributed to crystallization from the Zn/ZnO mixed phase to form the ZnO structure. As a result, such mixed phase processes can also explain why the ZnO nuclei grow into 1-D ZnO nanowires through short-circuit diffusion. As the annealing temperature increases, the thermal-enhanced surface diffusion occurs at the nodes of the ZnO nanowire, which are favored to form 3-D nanowires and nanoflowers at higher *T*_A_. This result is agreed with previous reported by Ng and co-authors for the growth of epitaxial ZnO nanowires [28].

## Conclusion

In summary, a diffusion ratio was obtained to deeply explore dimensionality-controllable synthesis of ZnO nanowires. For a lower dimensionality of ZnO nanowires, the *γ* value closes to 0.26(6), revealing a short-circuit diffusion mechanism. However, this tends to have a higher value of 0.41(3) as a further increase of annealing temperature results in the formation of 3-D ZnO nanowire network through lattice diffusion mechanism. These findings help us to proceed the fabrication of other novel nanostructure materials at various dimensionalities and the application in energy storage or memory devices through this growth mechanism.

## Endnotes

^a^The log-normal distribution is defined as follows:

(2) $$f(d)=\frac{1}{\sqrt{2\pi }d\sigma }\exp\left(-\frac{{\left(\ln d-\ln<d>\right)}^{2}}{2\sigma^{2}}\right)\text{,}$$

Where <*d*> is the mean value, and *σ* is the standard deviation of the function. ^b^The vibrational frequency of a Zn atom is defined as follows:

(3) $$v_{\text{D}}=\frac{1}{\sqrt{2}}{\left[\frac{Q_{\text{D}}}{m\alpha^{2}}\right]}^{\frac{1}{2}}\text{,}$$

where *α* = 0.285 nm is the *d*-spacing of the [100] plane; *m* = 65.4 g/mol, the Zn molar weight; and *Q*_D_ = 318 kJ/mol, the activation energy of Zn.

## Competing interests

The authors declare that they have no competing interests.

## Authors’ contributions

SYW wrote, conceived, and designed the experiments. PSS and HJH grew the samples and analyzed the data. YRM contributed the experimental facility of FE-SEM and valuable discussions. All authors discussed the results, contributed to the manuscript text, commented on the manuscript, and approved its final version. All authors read and approved the final manuscript.
